# Frequency of migraine in patients with allergic rhinitis

**DOI:** 10.12669/pjms.292.3148

**Published:** 2013-04

**Authors:** Ayhan Ozturk, Yildiz Degirmenci, Burcu Tokmak, Abdurrahman Tokmak

**Affiliations:** 1 Ayhan Ozturk, Associate Professor, Duzce University School of Medicine, Neurology Department, Duzce, Turkey,; 2 Yildiz Degirmenci, Assistant Professor, Duzce University School of Medicine, Neurology Department, Duzce, Turkey,; 3 Burcu Tokmak, Specialist, Cerkezkoy Hospital, Ear Nose Throat Department, Tekirdag, Turkey.; 4 Abdurrahman Tokmak, Specialist, Cerkezkoy Hospital, Ear Nose Throat Department, Tekirdag, Turkey.

**Keywords:** Allergic rhinitis, Histamine, Nitric oxide, Migraine

## Abstract

***Objectives:*** Allergic rhinitis (AR) is an IgE mediated disease which is released by activation of mast cells and basophils, and often leads to sinus headache. Histamine which is the key mediator in the pathogenesis of AR, also plays an important role in the pathogenesis of migraine with nitric oxide (NO). Aim of our study was to investigate the frequency of migraine in patients with AR.

***Methodology:*** Headache assessment and neurological examination was performed on patients diagnosed as AR in the outpatient ear nose and throat clinic with age-matched controls. Participants with headache were classified according to the International Headache Society criteria, and migraine frequency was investigated in the patients with AR and control groups.

***Results:*** Migraine headache was detected in 50% of the patients with AR. Among these, 95% were migraine without aura, and 5% were migraine with aura. Migraine frequency in the control group was 18.75% in the control group, and all was migraine without aura. Migraine frequency in the patients with AR was four times higher when compared with the control group.

***Conclusion:*** While a histamine and IgE associated common mechanism is responsible in the pathogenesis of AR and migraine, not only sinus headache but also migraine headache should be kept in mind. Headache assessment of the patients with AR, and in case of headache existence, referral of these patients to neurology outpatient clinics for differential diagnosis and, to maintain appropriate therapy should not be forgotten.

## INTRODUCTION

Allergic rhinitis is a chronic disease of seasonal and/or perennial course, characterized with antibody immunoglobulin E (IgE) mediated inflammation of nasal mucosa triggered by allergens including pollens, dust, foods, mites, animal dander, occupational allergens and drugs.^1^ European prevalence of the disease varies between 17% and 29%^2^, while the reported prevalence rate in Turkey is 17.5 %.^3^ AR is an allergic disease in which production of the antibody IgE as a response to the trigger allergens is the key in the pathogenesis of the disease. As soon as the Ig E binds to mast cells and basophils containing histamine and other chemicals, the release of these inflammatory mediators cause acute symptoms as sneezing, itchy and watery eyes, swelling and inflammation of the nasal passages, and an increase in mucus production. Late phase occurs 4-8 hours after the exposure to allergen and is characterized with additional symptoms as fatigue, irritability, and headache.^3^^-^^5^

Most common form of headache in patients with AR is sinus headache, which is caused by the activation of mast cells and basophils.^6^ Nitric oxide (NO) is another inflammatory mediator seen in AR, and is considered to play a role in migraine attacks due to its’ vasodilator effects.^7^^,^^8^ Migraine is a combination of autonomic, gastrointestinal, and neurological symptoms which is characterized with severe headaches. Reported prevalence of migraine in Turkey is 16.1%.^9^ Migraine is considered as a result of neurovascular process triggered by endogenous and/or exogenous factors in genetically predisposed individuals. However, pathogenesis and molecular mechanisms of migraine is still unclear.^10^

Underlying pathophysiological mechanisms include neurogenic inflammation, defects in the metabolism of arachidonic acid and serotonin, cyclic changes in the ovarian steroid concentrations, food allergy, and atopy. These mechanisms are important in assessing the relationship of migraine with allergic diseases.^11^ Additionally, it is a well-known fact that plasma histamine levels increase during both the headache and remission phase of migraine.^5^^,^^12^ While local histamine release in AR may trigger migraine attacks in these patients, we aimed to assess the frequency of migraine in patients with AR in our study.

## METHODOLOGY

Patients with the diagnoses of AR were included from the Ear Nose and Throat outpatient clinic of Duzce University School of Medicine between July and October 2007. Control group consisted of age-matched healthy individuals. All participants were informed about the content of the study and gave their approval before enrollment.

Exclusion criteria were sinusitis, upper respiratory infection, stroke, cerebral palsy, trigeminal neuralgia, epilepsy and allergic diseases other than AR. Clinical diagnosis was confirmed with skin tests and serum IgE levels in patients with AR. Skin test (Prick test-Allergopharma- Germany) was performed on the forearm of the patients with 16 solutions. Skin tests included solutions with histamine (positive control), physiological saline (negative control), and 14 allergenic substances. Diameter of the induration which existed in the upper layer of reaction was measured 15 minutes after the application of solutions and scored.^13^

All patients and controls underwent a detailed neurological and headache assessment following neurological examination. Participants with headache were re-evaluated and classified according to the criteria of International Headache Society (IHS), and the frequency of migraine was investigated.^14^

Data were organized in an SPSS version 11.0 (Statistical Package for Social Sciences for Windows) database. Values were given as mean ± standard deviations, and p values < 0.05 were considered to indicate statistical significance. χ2 test was used in the analysis of categorical variables, and numeric variables were compared with student’ s t test.

## RESULTS

Eighty AR patients with positive skin test were included in the study. Among these, three patients were under desensitization therapy, 77 were newly diagnosed patients without any treatment. Study group consisted of 50 female (62.5%) and 30 male (37.5 %) patients with AR. There were 46 female (57.5%) and 34 male (42.5%) participants in the control group. Mean age of the patients were 32.27 ± 12.50 years (range=12-62), and 34.06 ± 12.79 years (range=15-65) in the control group. There were no statistically significant difference between the age and gender of the study groups (p = 0.51, x² = 0.41 and p = 0.37, t = - 0.89, respectively). Socio-demographic features and migraine characteristics and frequencies of the study groups were summarized in Table-I.

Skin tests of the patients with AR revealed allergy to acar 1-2 in 68 of the patients (85%), to fungi 1-2 in 5 (6.25%), to mix grass in 4 (5%) and to grass and grain in three of the patients (3.75%). Among these, 40 patients were diagnosed as migraine (50%). Thirty eight of them were diagnosed as classical migraine without aura (95%), and two had migraine with aura (5%). Fifteen of the participants were diagnosed as migraine in the control group, and all was (were) classical migraine without aura (18.75%).

**Table-I:** Socio-demographic features and migraine characteristics of the study groups

	*Allergic rhinitis* *Number (%)*	*Control* *Number (%)*	*P* ^*^
Gender			
Female	50 (62,5)	46 (57,5)	
Male	30 (37,5)	34 (42,5)	
Total	80 (100)	80 (100)	0.51
Age			
12-20 years	12 (15)	4 (5)	
21-40 years	50 (62,5)	58 (72,5)	
41-65 years	18 (22,5)	18 (22,5)	
Total	80 (100)	80 (100)	0.37
Migraine			
With aura	2 (2,5)	0 (0)	
Without aura	38 (47,5)	15 (18,8)	
Without	40 (50)	65 (81,3)	
Total	80 (100)	80 (100)	<0.001

^*^p<0.05; statistically significant

**Table-II:** Migraine frequency according to gender in study groups

		*Migraine*				*Total (%)*
*Allergic rhinitis *	*Cinsiyet*	*With aura (%)*	*Without aura (%)*	p*	*Yok (%)*	
With	Kadin	2 (4)	28 (56)	0.0003	20 (40)	50 (100)
	Erkek	-	10 (33.3)	0.0037	20 (66.6)	30 (100)
	Toplam	2 (2.5)	38 (47.5)		40 (50)	80 (100)
Without	Kadin	-	11 (23.9)		35 (76)	46 (100)
	Erkek	-	4 (11.7)		30 (88.2)	34 (100)
	Toplam	-	15 (18.7)		65 (81.2)	80 (100)

^*^ p<0.05; statistically significant

Migraine frequency according to the gender in both groups is summarized in Table-II. Migraine frequency in females in the patients with AR was found to be increased when compared with the females in the control group (p = 0.0003). Similarly, migraine frequency in males in the patients with AR was found to be increased when compared with the males in the control group (p =0.0037). Frequency of migraine was found to be increased in the patients with AR, when compared with the control groups (50 ±0.098 and 18.8 ± 0.07, respectively), and this difference was statistically significant (p<0.001).

## DISCUSSION

Migraine is a primary episodic headache disorder with various neurological complaints. Previous reports revealed that endogenous molecules such as calcitonin gene related peptide, NO, and histamine play an important role in the pathophysiology of migraine. However, the exact underlying mechanism is still unclear.^15^^,^^16^

In AR, histamine is considered to contribute the development of migraine headaches via increasing the release of NO. Additionally, it facilitates the evolution of local neurogenic inflammation by increasing vasodilatation and vascular permeability via H1 and H2 receptors.^5^ Thus, immune response characterized with histamine and NO mediated inflammation is considered as the fundamental factor of AR pathogenesis.^1^^,^^7^

With respect to this common pathogenic mechanism in AR and migraine, we aimed to evaluate the migraine frequency in patients with AR. Our literature review revealed limited number of reports demonstrating the association between AR and migraine.^5^^,^^15^ But differing from the first study evaluating migraine frequency in patients with AR,^5^ we also investigated the triggering allergens in our study. Min Ku et al, reported that Migraine frequency was 34% in the patients with AR, while this rate was 4% in the control group.^5^ Similarly, we also found an increased frequency of migraine in patients with AR, when compared with the control group (50% and 18.75%, respectively), and this difference between the groups was statistically significant (p<0.05). When we evaluated the migraine frequency according to the gender difference between the groups, we found an increased rate of migraine frequency in women and men in the AR group.

There are several reports revealing the relationship of migraine with allergic diseases. However, the number of studies assessing migraine in AR is limited.^11^^,^^16^^,^^17^ Davey at al. emphasized the association between migraine and bronchial asthma,^11^ while Mortimer et al. reported a higher prevalence of migraine in children with atopy, when compared with children without atophy(atopy).^16^ A previous study evaluating the possible effects of migraine on allergy investigated the serum IgE and histamine levels during migraine attacks and remission periods in migraine patients with allergy and without allergy. Serum IgE and histamine levels were found to be increased in migraine patients with and without allergy, both when compared with healthy controls. Moreover, there was a statistically significant difference between migraine patients with and without allergy, in which migraine patients with allergy had significantly higher serum IgE and histamine levels. Similar to our results, Gazerani et al. suggested a possible IgE mediated common mechanism in the pathophysiology of migraine and allergy.^18^

In conclusion, results of our study were compatible with the hypothesis of IgE and histamine mediated common mechanism in migraine and AR. Thus, it is important to keep in mind that migraine headaches can be seen in patients with AR, as well as sinus headaches. As such AR patients with the complaint of headache should be referred to neurology outpatient clinics for differential diagnosis and appropriate treatment of headache. In such patients, future studies evaluating the effects of AR treatment in migraine headache may be beneficial in enhancing the treatment options, as well.
